# Supplementary material for: Genetic regulators of neuronal survival across metabolic environments
Source: bioRxiv. 2025 Dec 19:2025.12.19.695350. Preprint. [Version 1] doi: 10.64898/2025.12.19.695350 (PMC12880232; doi:10.64898/2025.12.19.695350)
Supplement: Supplement 1 [file NIHPP2025.12.19.695350v1-supplement-1.pdf]

bioRxiv preprint doi: https://doi.org/10.64898/2025.12.19.695350; this version posted December 19, 2025. The copyright holder for this preprint (which was not certified by peer review) is the author/funder, who has granted bioRxiv a license to display the preprint in perpetuity. It is made available under aCC-BY 4.0 International license.

Supplementary Figure Legends

Supplementary Fig 1. A) STRING-based physical interactions combined with survival screen phenotypes nominate important roles for β-arrestin (encoded by gene *ARBB1*) and specific olfactory receptors OR6K3 and OR5M10 in regulating neuronal survival in hypoxic conditions with unrestricted metabolism. Similarly, this analysis nominates activity of the SCF E3 ubiquitin ligase complex, which depends on NEDD8 and CDC34, as essential for neuron survival in hypoxic conditions, with either unrestricted metabolism or when metabolism is restricted to respiration-only. B) Knockdown of few glycolytic enzyme-encoding genes lead to decreased glycolytic survival when neuronal cells are forced to rely on glycolysis-only in high oxygen conditions. Glycolytic enzymes are colored based on their log-fold change in enrichment of their associated targeting sgRNA in the genome-wide CRISPRi screen post-shift in metabolic conditions versus pre-treatment. B) Knockdown of glycolytic enzyme-encoding genes also did not have strong effects on survival when neuronal cells are forced to rely on respiration-only. Data for genome-wide CRISPRi screens compiled from n= 2 screens. C) Neuronal cells forced to rely on respiration-only incorporated significantly more carbons derived from 13C-pyruvate into lactate and TCA cycle metabolite pools, compared to neuronal cells cultured in unrestricted metabolism conditions. However, neuronal cells forced to rely on respiration-only had total TCA metabolite

bioRxiv preprint doi: https://doi.org/10.64898/2025.12.19.695350; this version posted December 19, 2025. The copyright holder for this preprint (which was not certified by peer review) is the author/funder, who has granted bioRxiv a license to display the preprint in perpetuity. It is made available under aCC-BY 4.0 International license.

pool sizes that were not significantly different than in unrestricted metabolism conditions, except that they had significantly smaller malate pool, increased total levels of purine metabolites with fewer high-energy phosphate bonds (AMP, GMP), and significantly smaller total levels of nucleotides with more high-energy phosphate bonds (UTP, ATP). *$p < 0.05$, **$p < 0.01$, ***$p < 0.001$, ****$p < 0.0001$ by 2-way ANOVA with Šídák's multiple comparisons test.

Supplementary Fig 2. A) *MAPT* knockdown was not associated with decreased neuronal survival in unrestricted, physiologic, or respiratory survival when targeted as part of a genome-wide dual-guide CRISPRi library or a targeted single-guide mini CRISPRi library. Dashed lines depict 2 standard deviations from the average of non-targeting guides in each screen. Data is compiled from n = 2 replicates per metabolic state for the genome-wide screen, n = 4 replicates for unrestricted and untreated metabolic samples, n = 6 replicates for respiratory, physiologic, and glycolytic metabolic state for the mini-library screen. B) Loading for *MAPT* and *RRAD* highlight that the variation *MAPT* expression correlates with the variation across metabolic states examined in the genome-wide CRISPRi screen along the first principal component. Notably, the first principal component explains variation across glycolytic states across the range of oxygen levels. *RRAD* expression correlates with variation across metabolic states along the second principal component, which accounts for separation between glycolytic and non-glycolytic metabolic states. C) Neuronal cells with knockdown in *MAPT* expression have no significant change in 13C-glucose derived labeling in high-oxygen glycolytic conditions, compared to neuronal cells expressing a non-targeting sgRNA, with the exception of increased 13C-glucose derived labeling in the glucose-6-phosphate/fructose-6-phosphate metabolite pool. Data compiled from n = 4 samples. ****$p < 0.0001$ by 2-way ANOVA with Šídák's multiple comparisons test. D) Neuronal cells expressing the *MAPT*-targeting CRISPRi sgRNA in the Tian et. al perturb-seq dataset[43] do not have significant alterations in expression of glycolytic transcripts.

Supplementary Fig 3. Individually created neuronal cells lines have robust CRISPRi-mediated knockdown of targeted genes *MAPT*, *KIAA1429*, *GLO1*, and *TDRD3*, measured by RT-qPCR. Data compiled from n = 2 independent replicates. ****$p < 0.0001$ by t-test with Welch's correction.

Supplementary Fig 4. A) Aerobic glycolysis-related genes feature among the top 5 pathways in Human Wikipathways database, based on enrichment score, which were over-represented among the most co-downregulated genes across CRISPR-perturbations with significantly downregulated *KIAA1429* expression, compiled from the Tian et. al. 2019 and Tian et. al. 2021 perturb-seq datasets[9, 43]. B) Aerobic glycolysis-related genes also feature among the top 5 pathways in Human Wikipathways database, based on enrichment score, which were over-represented among downregulated genes in neurons with *KIAA1429* knockdown compared to neurons expressing a non-targeting sgRNA. All depicted pathways have an adjusted P-value < 0.05.

Supplementary Fig 5. Neuronal cells with knockdown of genes essential for survival when forced to rely on glycolysis-only in high oxygen conditions (*MAPT, KIAA1429, TDRD3*) divert carbons

bioRxiv preprint doi: https://doi.org/10.64898/2025.12.19.695350; this version posted December 19, 2025. The copyright holder for this preprint (which was not certified by peer review) is the author/funder, who has granted bioRxiv a license to display the preprint in perpetuity. It is made available under aCC-BY 4.0 International license.

from 13C-glucose to nucleotides (AMP, ADP, UDP), compared to neuronal cells expressing a non-targeting sgRNA. Knockdown of a less functionally related gene, *GLO1*, does not result in increased diversion of carbons derived from 13C-glucose to nucleotides, and actually decreases labeling in ADP and UDP metabolite pools. Data compiled from n = 3-4 samples per condition. *$p < 0.05$, **$p < 0.01$, ****$p < 0.0001$ by 2-way ANOVA with Šídák's multiple comparisons test.
